# Supplementary material for: From sequence to enzyme mechanism using multi-label machine learning
Source: BMC Bioinformatics. 2014 May 19;15:150. doi: 10.1186/1471-2105-15-150 (PMC4229970; doi:10.1186/1471-2105-15-150)
Supplement: Additional file 2 — Java code of ml2db. Additional file ml2db_code.tar.gz contains the Java source code to run the multi-label machine learning experiments and save the results to database. The code’s Javadoc is included. [file 1471-2105-15-150-S2.zip › additional file 2/ml2db/utils/doc/constant-values.html]

Constant Field Values


JavaScript is disabled on your browser.


- Overview
- Package
- Class
- Use
- Tree
- Deprecated
- Index
- Help

- Prev
- Next

- Frames
- No Frames

- All Classes

# Constant Field Values

## Contents

- cern.jet.\*
- edu.cornell.\*
- uk.ac.\*

## cern.jet.\*

- cern.jet.random.engine.MersenneTwister

  | Modifier and Type | Constant Field | Value |
  | --- | --- | --- |
  |  |  |  |
  | --- | --- | --- |
  | `public static final int` | `DEFAULT_SEED` | `4357` |


## edu.cornell.\*

- edu.cornell.lassp.houle.RngPack.Ranlux

  | Modifier and Type | Constant Field | Value |
  | --- | --- | --- |
  |  |  |  |
  | --- | --- | --- |
  | `public static final int` | `lxdflt` | `3` |
  | `public static final int` | `maxlev` | `4` |


## uk.ac.\*

- uk.ac.standrews.utils.main.TimeUtils

  | Modifier and Type | Constant Field | Value |
  | --- | --- | --- |
  |  |  |  |
  | --- | --- | --- |
  | `public static final java.lang.String` | `DAY` | `"day"` |
  | `public static final java.lang.String` | `HOUR` | `"hour"` |
  | `public static final java.lang.String` | `MILLISECONDS` | `"milliseconds"` |
  | `public static final java.lang.String` | `MINUTE` | `"minute"` |
  | `public static final java.lang.String` | `MONTH` | `"month"` |
  | `public static final java.lang.String` | `SECOND` | `"second"` |
  | `public static final java.lang.String` | `YEAR` | `"year"` |

- uk.ac.standrews.utils.main.collectionutils.setutils.SupersetsManager

  | Modifier and Type | Constant Field | Value |
  | --- | --- | --- |
  |  |  |  |
  | --- | --- | --- |
  | `public static final boolean` | `m_debug` | `false` |

- uk.ac.standrews.utils.main.database.DbConn

  | Modifier and Type | Constant Field | Value |
  | --- | --- | --- |
  |  |  |  |
  | --- | --- | --- |
  | `public static final java.lang.String` | `DATABASE_NAME_PROP` | `"database_name"` |
  | `public static final java.lang.String` | `DATABASE_TYPE_PROP` | `"database_type"` |
  | `public static final java.lang.String` | `HOST_PROP` | `"host"` |
  | `public static final java.lang.String` | `JDBC_DRIVER_PROP` | `"jdbc_driver"` |
  | `public static final java.lang.String` | `PASSWORD_PROP` | `"pswd"` |
  | `public static final java.lang.String` | `PORT_PROP` | `"port"` |
  | `public static final java.lang.String` | `USER_PROP` | `"user"` |
- uk.ac.standrews.utils.main.database.DbUtils

  | Modifier and Type | Constant Field | Value |
  | --- | --- | --- |
  |  |  |  |
  | --- | --- | --- |
  | `public static final java.lang.String` | `AUTO_INCREMENT_SQL_DATATYPE` | `"INT AUTO_INCREMENT"` |
  | `public static final java.lang.String` | `BOOLEAN_SQL_DATATYPE` | `"BOOLEAN"` |
  | `public static final java.lang.String` | `CHAR_2_SQL_DATATYPE` | `"CHAR(2)"` |
  | `public static final java.lang.String` | `CHAR_4_SQL_DATATYPE` | `"CHAR(4)"` |
  | `public static final java.lang.String` | `CHAR_6_SQL_DATATYPE` | `"CHAR(6)"` |
  | `public static final java.lang.String` | `DOUBLE_QUOTE_CHARACTER` | `"\""` |
  | `public static final java.lang.String` | `DOUBLE_SQL_DATATYPE` | `"DOUBLE"` |
  | `public static final java.lang.String` | `END_TIMESTAMP` | `"end_time"` |
  | `public static final java.lang.String` | `ID` | `"id"` |
  | `public static final java.lang.String` | `INTEGER_SQL_DATATYPE` | `"INTEGER"` |
  | `public static final java.lang.String` | `LONG_TEXT_SQL_DATATYPE` | `"LONGTEXT"` |
  | `public static final long` | `MAX_FILE_SIZE` | `8000000L` |
  | `public static final java.lang.String` | `REVERSED_PRIME_CHARACTER` | `` "`" `` |
  | `public static final java.lang.String` | `SINGLE_QUOTE_CHARACTER` | `"\'"` |
  | `public static final java.lang.String` | `START_TIMESTAMP` | `"start_time"` |
  | `public static final java.lang.String` | `TEXT_SQL_DATATYPE` | `"TEXT"` |
  | `public static final java.lang.String` | `TIMESTAMP` | `"timestamp"` |
  | `public static final java.lang.String` | `TIMESTAMP_DOWNLOADED` | `"time_downloaded"` |
  | `public static final java.lang.String` | `TIMESTAMP_SQL_DATATYPE` | `"TIMESTAMP"` |
  | `public static final java.lang.String` | `VARCHAR_16_SQL_DATATYPE` | `"VARCHAR(16)"` |
  | `public static final java.lang.String` | `VARCHAR_255_SQL_DATATYPE` | `"VARCHAR(255)"` |
  | `public static final java.lang.String` | `VARCHAR_26_SQL_DATATYPE` | `"VARCHAR(26)"` |
  | `public static final java.lang.String` | `VARCHAR_90_SQL_DATATYPE` | `"VARCHAR(90)"` |
- uk.ac.standrews.utils.main.database.SqlUtils

  | Modifier and Type | Constant Field | Value |
  | --- | --- | --- |
  |  |  |  |
  | --- | --- | --- |
  | `public static final int` | `DOUBLE` | `2` |
  | `public static final int` | `INTEGER` | `1` |
  | `public static final int` | `MAX_BIGINT_LENGTH` | `18` |
  | `public static final int` | `MAX_CHAR_LENGTH` | `255` |
  | `public static final int` | `MAX_INT_LENGTH` | `9` |
  | `public static final int` | `NULL` | `0` |
  | `public static final int` | `STRING` | `3` |

- uk.ac.standrews.utils.main.diff.Difference

  | Modifier and Type | Constant Field | Value |
  | --- | --- | --- |
  |  |  |  |
  | --- | --- | --- |
  | `public static final int` | `NONE` | `-1` |

- uk.ac.standrews.utils.main.setutils.SupersetsManager

  | Modifier and Type | Constant Field | Value |
  | --- | --- | --- |
  |  |  |  |
  | --- | --- | --- |
  | `public static final boolean` | `m_debug` | `false` |

- uk.ac.standrews.utils.test.Data

  | Modifier and Type | Constant Field | Value |
  | --- | --- | --- |
  |  |  |  |
  | --- | --- | --- |
  | `public static final java.lang.String` | `KEY1` | `"key1"` |
  | `public static final java.lang.String` | `KEY2` | `"key2"` |
  | `public static final java.lang.String` | `KEY3` | `"key3"` |
  | `public static final java.lang.String` | `KEYA` | `"keyA"` |
  | `public static final java.lang.String` | `KEYB` | `"keyB"` |
  | `public static final java.lang.String` | `KEYC` | `"keyC"` |
  | `public static final java.lang.String` | `VAL1` | `"value1"` |
  | `public static final java.lang.String` | `VAL2` | `"value2"` |
  | `public static final java.lang.String` | `VAL3` | `"value3"` |
  | `public static final java.lang.String` | `VALA` | `"valueA"` |
  | `public static final java.lang.String` | `VALB` | `"valueB"` |

- uk.ac.standrews.utils.test.database.TableCreatorTest

  | Modifier and Type | Constant Field | Value |
  | --- | --- | --- |
  |  |  |  |
  | --- | --- | --- |
  | `public static final java.lang.String` | `TABLE1` | `"table1"` |

- uk.ac.standrews.utils.test.stats.TruncatedParetoTest

  | Modifier and Type | Constant Field | Value |
  | --- | --- | --- |
  |  |  |  |
  | --- | --- | --- |
  | `public static final int` | `THRESHOLD_100` | `100` |

- Overview
- Package
- Class
- Use
- Tree
- Deprecated
- Index
- Help

- Prev
- Next

- Frames
- No Frames

- All Classes
